# The Effect of Integrity of Lateral Wall on the Quality of Reduction and Outcomes in Elderly Patients with Intertrochanteric Fracture: A Controlled Study

**DOI:** 10.1155/2021/6563077

**Published:** 2021-08-09

**Authors:** Hong-Li Deng, Yu-Xuan Cong, Hai Huang, Bin-Fei Zhang, Ya-Hui Fu, Jin-Lai Lei, Hu Wang, Peng-Fei Wang, Yan Zhuang, Chao Ke

**Affiliations:** Department of Orthopedic Trauma, Honghui Hospital, Xi'an Jiaotong University, Xi'an, Shaanxi Province, China

## Abstract

**Objective:**

The study is aimed at evaluating the effect of the integrity of lateral wall on the quality of reduction and outcome in intertrochanteric fracture treated with proximal femoral nail antirotation (PFNA).

**Methods:**

Medical record systems for elderly patients with intertrochanteric fracture treated with PFNA were included. The patients were divided into incompetent and intact lateral wall groups. Patients' baseline characteristics, quality of reduction, and Harris Hip scores (HHS) were collected.

**Results:**

The study included 115 patients with intertrochanteric fractures, with 59 in the incompetent lateral wall group and 56 in the intact group. Lateral wall thickness was 16.47 ± 2.46 mm and 23.68 ± 1.59 mm in the incompetent group and intact group (*t* = −18.766, *P* < 0.001), respectively. There was no significant difference in the quality of reduction (*P* = 0.646) between intact and incompetent groups. Mean HHS at final follow-up were 83.02 ± 13.89 in the incompetent group and 86.04 ± 3.39 in the intact group, with no significant difference (*P* = 0.123). In addition, there was no significant difference in weight-bearing or clinical healing between intact and incompetent groups. The partial weight-bearing with crutches was allowed at 2.71 ± 0.93 and 2.66 ± 1.01 weeks after the operation in the incompetent and intact groups. Time to clinical healing was 5.83 ± 0.99 and 6.00 ± 0.92 months in the incompetent and intact groups, respectively. However, the operative time in the incompetent group (58.54 ± 18.14 mins) were longer than that in the intact group (51.79 ± 17.77 mins).

**Conclusions:**

In conclusion, it seems that lateral wall thickness does not affect the quality of reduction and outcome in patients with intertrochanteric fracture receiving PFNA.

## 1. Introduction

Geriatric intertrochanteric fractures represent an increasing public health problem all over the world. According to recent epidemiological investigation, the incidence rates of intertrochanteric fractures were 171/100,000 and have been kept increasing recently [1].

The trochanteric lateral wall is primarily the lateral femoral cortex of the drilling site for head-neck fixation implant [2]. Describing in Hsu's study, the lateral wall thickness is the distance in millimeters from a reference point 3 cm below the innominate tubercle of the greater trochanter, angled 135° upward to the fracture line on the anteroposterior radiograph [3]. This criterion has been accepted and included in the 2018 version of AO/OTA Fracture and Dislocation Classification Compendium [4]. If the thickness is less than 20.5 mm, the lateral wall is divided into incompetent subtype.

The intact lateral wall plays a crucial role in the stabilization of intertrochanteric fracture. Under the implant of extramedullary fixation, an intact lateral wall could provide good biomechanical support [5, 6]. However, intramedullary nailing is a good option, especially when lateral wall is incompetent [7]. Therefore, the integrity of the lateral wall in intertrochanteric fractures plays an essential role in choosing the type of implant. In the past, the proximal femoral locked compression plate was recommended for use in fixation of complex proximal femur fractures such as A3 intertrochanteric fracture in AO/OTA classification version 2007 [8]. However, rates of failure after fixation using plate for those fractures were reported as 44% [9], and the reoperation rate (4%) after surgery with intramedullary nailing was significantly lower than that after surgery with sliding hip screw [10]. In addition, the strength of lateral wall in elderly people is so weak, and the preoperatively intact lateral wall may be broken during the operation when using plate in some patients. Under these views, some surgeons prefer intramedullary nails in all types of intertrochanteric fractures.

The intact lateral wall takes about 67% of all intertrochanteric fractures, and incompetent lateral wall takes 33% [11]. Duration of the operation, intact lateral wall demonstrates the simple fracture lines, and it is easy to reduce. In contrast, incompetent lateral wall is usually difficult to achieve good reduction because of multifragmentary fractures. Even though the new AO/OTA classification was introduced in 2018, there are no related studies involving the quality of reduction and prognosis affected by the lateral wall. Thus, the present study is aimed at evaluating the effect of the integrity of lateral wall on the quality of reduction and outcome in intertrochanteric fracture treated with intramedullary nails.

## 2. Materials and Methods

### 2.1. Patients including

The Ethics Committee of the Xi'an Honghui Hospital approved the study (No. 2020064). The inclusion criteria were (1) age ≥ 65 years; (2) hip pain, tenderness, dysfunction, ecchymosis, and local swelling; (3) injury from falling from a height, slipping, traffic accident, or other; (4) unilateral intertrochanteric fractures were confirmed using radiography, and the integrity of lateral wall could be distinguished; (5) operative treatment of closed reduction and internal fixation was undergone by proximal femoral nail antirotation (PFNA); and (6) at least six months of follow-up.

**Figure 1:**
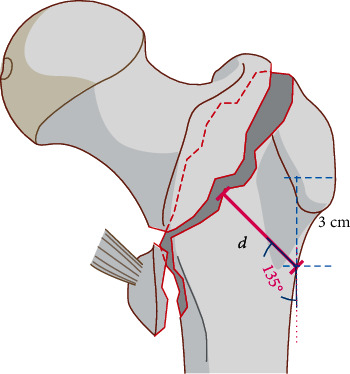
The schematic diagram of the intertrochanteric lateral wall.

The exclusion criteria were (1) age < 65 years, (2) multiple injuries with intertrochanteric fractures and other fractures and only operation for intertrochanteric fractures, (3) severe comorbidities and could not suffer the operation, and (4) the patients received the extramedullary hip screws. We searched the medical system records for patients with intertrochanteric fractures. The patient record search periods were from January 2018 to June 2019. In Figure 1, when the thickness (*d*) was less than 20.5 mm, it was divided into incompetent group; when (*d*) was longer than 20.5 mm, it was divided into intact group.

### 2.2. Surgical Strategy

The surgical strategy is similar to the article from Ma et al. [12]. Upon admission of patients with intertrochanteric fractures, the blood routine test and other blood samples were examined to assess the hidden blood loss. When patients were in stable condition, we performed the operation as soon as possible. All procedures were performed under general anesthesia by the same team.

Duration the operation, the C-arm fluoroscopy was used to check the reduction and the procedure of inserting the nail. We used the anterior-posterior and lateral views of the hip to assess the quality of reduction. We tried the closed reduction firstly. If the quality is poor, there would be another reduction. Once the reduction was acceptable, we used the guide needle to locate the insertion point by puncturing the skin. After reconfirming the fracture reduction, we cut a 5 cm incision along the direction of the guide needle. The proximal femur was gradually reamed, and then, a nail was implanted. After adjusting the height of the nail, the frame to implant the head screw was fixed. After inserting the needle and measuring the length of the head screw and reaming, the head screw was implanted and compressed the fracture. Then, a distal locking screw was implanted under the directing frame, and a tail screw of the nail was locked. Finally, we washed out and closed the incision.

### 2.3. Follow-Up

After discharging, the patients were recommended to perform isometric exercises in bed as soon as possible and allowed partial weight-bearing at 2-4 weeks postoperatively. The surgeons determined the timing of full weight-bearing according to fracture healing. The patients returned to the hospital at least once a month for the first six months postoperatively, and an X-ray was used to evaluate the fracture union.

### 2.4. Outcomes

The outcomes were quality of reduction, Harris Hip scores (HHS), operative time, intraoperative blood loss, blood transfusion, intraoperative liquid, follow-up time, clinical healing time, weight-bearing time, and complications (deep vein thrombosis, wound infection, revision, mortality). Chang's reduction quality criteria was used as the tool to assess the quality of reduction [13, 14].

### 2.5. Statistical Analysis

Statistical analysis was performed using SPSS version 25.0 (SPSS Inc., Chicago, IL, USA). We assessed whether measurement data were normally distributed using the Shapiro-Wilk test and then using independent-samples *t*-tests. For frequency data, the chi-square or Fisher's exact test was used. If *P* was <0.05, there was the considered statistically significant difference.

## 3. Results

### 3.1. Clinical Characteristics

This study included 115 patients who suffered intertrochanteric fractures. There were 82 females and 33 males. The lateral wall incompetent group included 59 patients, and the intact group included 56 patients admitted between January 2018 and June 2019. The same team performed all 115 operations. The mean age was 80.07 years in the incompetent group and 80.63 years in the intact group, respectively. Mechanisms of injury included slipped, high falling, accident, and others. The most common mechanism was slipped, which occurred in 81.35% and 89.28% of patients in incompetent and intact groups, respectively. Preoperative Visual Analogue Scale (VAS) scores were 3.86 ± 0.99 and 4.25 ± 1.15 in the incompetent and intact groups, respectively.

Lateral wall thickness was 16.47 ± 2.46 mm and 23.68 ± 1.59 mm in the incompetent group and intact group (*t* = −18.766, *P* < 0.001), respectively. The intertrochanteric fractures were divided into three subgroups according to the 2007 AO/OTA classification, and there were 1, 28, and 30 of the incompetent group and 23, 17, and 16 of the intact group in 31A1.3, 31A2.2, and 31A2.3 subgroups, respectively. We found a statistically significant difference in fracture types between the two groups (*χ*^2^ = 27.057, *P* < 0.001). The percentage of 31A1.3 (41%) in the intact group was more than the incompetent group (1.6%). In addition, there were no significant differences in the comorbidities (hypertension, diabetes, stroke, and associated injuries) between the two groups. The time from injury to admission was 0.97 ± 0.83 and 0.86 ± 0.86 days in the incompetent group and intact group, respectively. The time from admission to operation was 2.53 ± 0.95 days and 2.27 ± 0.86 days in an incompetent and intact group. The length of stay in hospital in the incompetent group (7.39 ± 3.40 days) was similar to the intact group (6.52 ± 2.35 days). Detailed baseline information is shown in Table 1.

### 3.2. Comparison of Operative Characteristics

Mean operative time were 58.54 ± 18.14 mins and 51.79 ± 17.77 mins in the incompetent and intact groups, respectively. The operative time in the incompetent group was longer than that in the intact group (*t* = 2.016, *P* = 0.046). However, the blood transfusion in the incompetent group (1.31 ± 1.16 U) was similar to that in the intact group (1.29 ± 1.11 U, *P* = 0.908). The intraoperative blood loss was 64.75 ± 19.51 ml in the incompetent group and 68.21 ± 22.89 ml in the intact group, without significant differences. Also, there was no significant difference in the intraoperative liquid between the two groups (*P* = 0.059), incompetent group (1305.08 ± 302.53 ml) versus intact group (1203.57 ± 266.26 ml; Table 2).

### 3.3. Follow-Up and Fracture Healing

**Figure 2:**
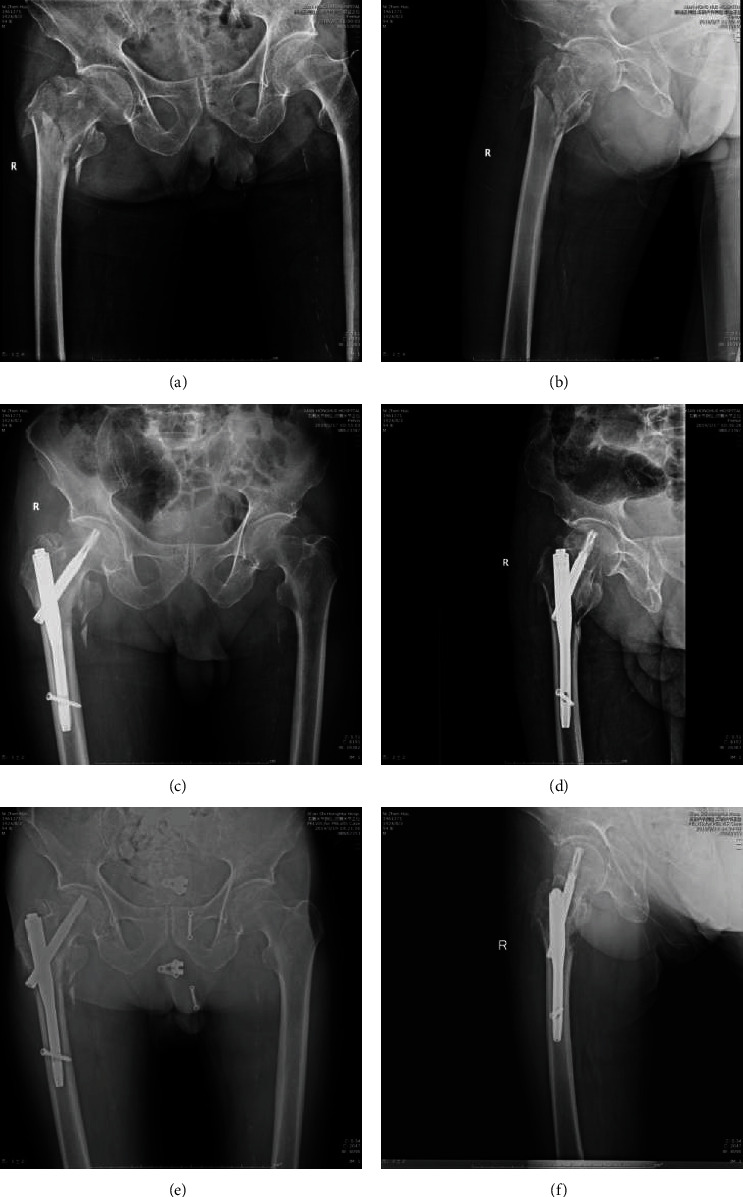
The patient receiving PFNA with incompetent lateral wall: (a) preoperative anterior-posterior view; (b) preoperative lateral view; (c) postoperative anterior-posterior view; (d) postoperative lateral view; (e) postoperative 3 months anterior-posterior view; (f) postoperative 3 months lateral view.

**Figure 3:**
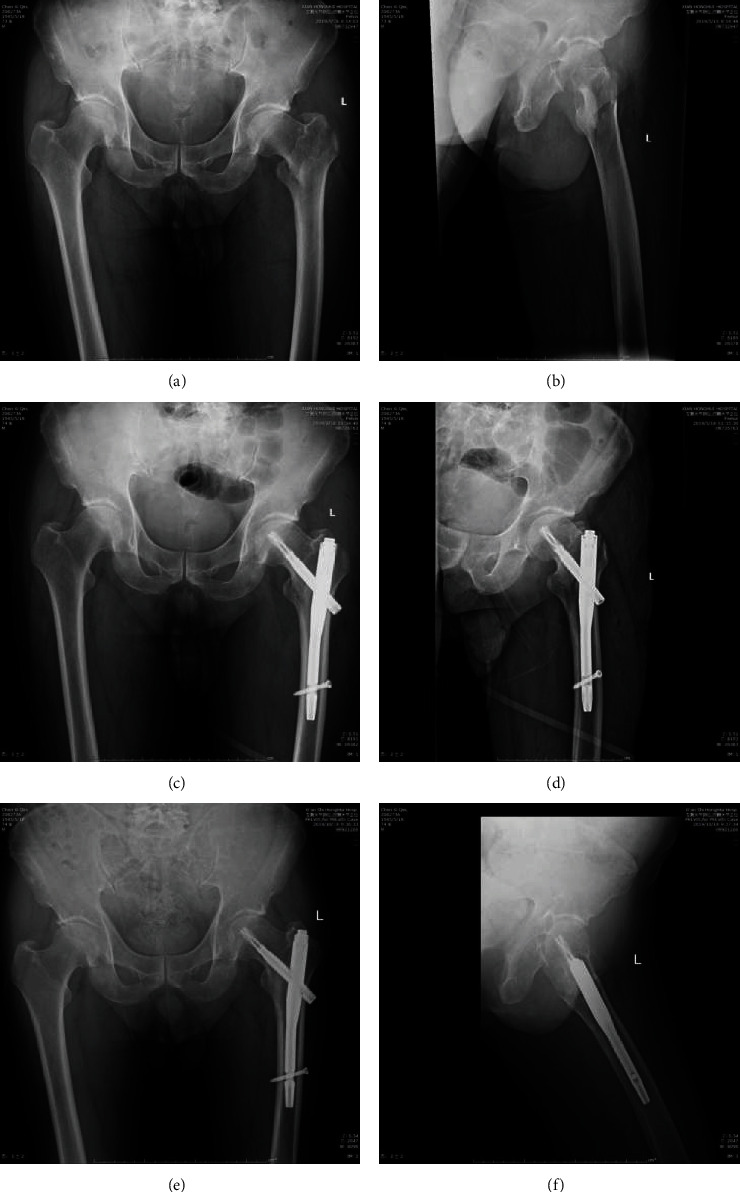
The patient receiving PFNA with intact lateral wall: (a) preoperative anterior-posterior view; (b) preoperative lateral view; (c) postoperative anterior-posterior view; (d) postoperative lateral view; (e) postoperative 4 months anterior-posterior view; (f) postoperative 4 months lateral view.

Follow-up time was not significantly different between the two groups (10.98 ± 4.32 months in the incompetent group and 11.13 ± 3.92 months in the intact group; *P* = 0.854). During the follow-up, we assessed the time to postoperative weight-bearing and time to clinical healing. The partial weight-bearing with crutches was allowed at 2.71 ± 0.93 and 2.66 ± 1.01 weeks after the operation in the incompetent and intact groups. We evaluated clinical healing based on radiographic findings, symptoms, and signs. Time to clinical healing was 5.83 ± 0.99 and 6.00 ± 0.92 months in the incompetent and intact groups, respectively. We found no significant differences between the two groups in the time to weight-bearing or clinical healing (Table 2). The patient with an incompetent lateral wall is shown in Figure 2, and with intact lateral wall is shown in Figure 3.

### 3.4. Functional Outcomes and Quality of Reduction

Mean HHS at final follow-up were 83.02 ± 13.89 in the incompetent group and 86.04 ± 3.39 in the intact group, with no significant difference (*P* = 0.123; Table 2). The quality of reduction was assessed after the operation by X-ray view. The quality was divided into three levels: excellent, acceptable, and poor. In the incompetent group, the excellent level has taken 54% of all, and the acceptable level has taken 42% of all. In the intact group, the excellent level has taken 62% of all, and the acceptable level has taken 34% of all. There were no significant differences in the distribution (*P* = 0.646; Table 2). The four patients who suffered a poor reduction were classified into 31A2.3 types.

### 3.5. Postoperative Complications

We assessed the deep vein thrombosis, superficial infection, revision, and mortality after the operation (Table 2). No deaths occurred during the hospital stay and following-up. In the incompetent and intact groups, 22 and 23 patients developed into deep vein thrombosis, respectively, with no significant difference in frequency (*P* = 0.678). One patient had the superficial infection in the intact group, and the patient was treated with antibiotics and wound care, and the infections ultimately healed. None of the patients experienced fixation failure needing revision during follow-up.

## 4. Discussion

The 2018 Compendium described it on the anteroposterior X-ray measuring the lateral wall height to identify the intact and incompetent subtypes [4]. In essence, the role of the mechanical buttress of the lateral wall is primarily played by the anterior or anteromedial cortex [15].

The present study is aimed at evaluating the quality of reduction and outcome in intertrochanteric fracture with intact and incompetent lateral wall. The results show that (1) there was no significant difference in the quality of reduction between in intact and incompetent group, (2) mean HHS at final follow-up was comparable in two groups, (3) the mean operative time in the incompetent group was longer than that in the intact group, and (4) there were no significant differences between the two groups in the time to weight-bearing or clinical healing.

In the distribution of fracture types, the percentage of 31A1.3 in the intact group was more than the incompetent group. This difference was depending on the characteristics of the integrity of lateral wall and the classification of version 2018 [4]. In this study, considering the unstable intertrochanteric fractures and the necessity of intramedullary fixation and the possibility of intraoperative or postoperative lateral wall fracture when using the extramedullary plates [7, 16], we restricted the included population as the receiving PFNA treatment to reduce the performance bias.

To our knowledge, this study is the first retrospective research focusing on the quality of reduction in intertrochanteric fractures. When the quality levels were divided into excellent, acceptable, and poor, we found that the excellent and acceptable levels taken 96% of all in the incompetent and intact groups, respectively. The assessing of reduction was used Chang's criterion, with a good receiver operating characteristic curve and 0.87 area under the curve [13, 14]. Two chief orthopedic surgeons assessed it independently, and the third chief surgeon would participate in assessment when encounter disagrees.

When to HHS comparison, it was comparable in two groups. The patients we included in the study were more than 65 years, and the mean HHS was 84.47. In a study from Korea [17], the HHS at 6 months is 88.33-90.68 in an average of 76 years. Overall, the function in these patients was good.

In the quality of reduction and HHS, we did not find the differences between incompetent and intact lateral wall groups. However, there is a trend that mean HHS in the intact group (86.04 ± 3.39) was higher than the incompetent group (83.02 ± 13.89), and the proportion of excellent level in the intact group (62%) was more than the incompetent group (54%), without the statistically significant difference.

In this study, the mean operative time in the incompetent group was longer than that in the intact group. Duration the operation, all the patients received the closed reduction. In the incompetent group, we often use one or two small incisions to assist in reducing with periosteal elevator or 90-degree pliers. Once the reduction was acceptable, we often choose a short 170 mm intramedullary nail, and the procedure of inserting the nail is the same in everyone patient. Thus, we consider that the difference in operative time is from the process of reduction, especially in the assisted incision in the incompetent group. In a prospective study, the mean operative time is 57.25 mins, which is similar to our result [17]. Corresponds to the operative time, the fluoroscopy time in the incompetent group is more than that in the intact group [17]. We did not compare this index in the study.

In addition, there were no significant differences between the two groups in the time to weight-bearing or clinical healing. The partial weight-bearing with crutches was allowed at 2-3 weeks after the operation in the incompetent and intact groups for these patients. The time to clinical healing was nearly six months in the incompetent and intact groups. Duration the follow-up, none of the patients experienced fixation failure.

In this controlled study, the results demonstrate that it is not so important to distinguish the integrity of the lateral wall when choosing intramedullary fixation, evaluating the quality of reduction or prognosis.

Indeed, there is a limitation in this study. The design of this study is retrospective single-center research; the patients in this study are divided into two groups according to the integrity of lateral wall. Although the baseline is comparable, there is the chance of selecting bias.

## 5. Conclusions

In conclusion, it seems that lateral wall thickness does not affect the quality of reduction and outcome in patients with intertrochanteric fracture receiving PFNA.

## Figures and Tables

**Table 1:** The characteristics in lateral wall incompetent group and lateral wall intact group.

	Lateral wall incompetent group	Lateral wall intact group	Total	*t*/*χ*^2^	*P*
No. of patients	59	56	115		
Age	80.07 ± 7.79	80.63 ± 6.14	80.34 ± 7.01	-0.427	0.670
Sex					
Female	38	44	82	2.817	0.093
Male	21	12	33		
Mechanism of injury					
Slipped	48	50	98	2.831	0.418
High falling	3	2	5		
Accident	5	1	6		
Other	3	3	6		
Preoperative VAS	3.86 ± 0.99	4.25 ± 1.15	4.05 ± 1.08	-1.924	0.057
Lateral wall thickness (mm)	16.47 ± 2.46	23.68 ± 1.59	19.98 ± 4.17	-18.766	<0.001
AO/OTA classification (2007)					
A1.3	1	23	24	27.057	<0.001
A2.2	28	17	45		
A2.3	30	16	46		
Comorbidities					
Hypertension	25	29	54	1.022	0.312
Diabetes	24	17	41	1.334	0.248
Stroke	20	17	37	0.165	0.685
Associated injuries	5	6	11	0.167	0.683
Days from injury to admission (days)	0.97 ± 0.83	0.86 ± 0.86	0.91 ± 0.84	0.691	0.491
Days from admission to operation (days)	2.53 ± 0.95	2.27 ± 0.86	2.40 ± 0.92	1.515	0.132
Length of stay in hospital (days)	7.39 ± 3.40	6.52 ± 2.35	6.97 ± 2.96	1.592	0.114

**Table 2:** The primary and secondary outcomes between two groups.

	Lateral wall incompetent group (*n* = 59)	Lateral wall intact group (*n* = 56)	Total	*t*/*χ*^2^	*P*
Operative time (mins)	58.54 ± 18.14	51.79 ± 17.77	55.34 ± 18.26	2.016	0.046
Intraoperative blood loss (ml)	64.75 ± 19.51	68.21 ± 22.89	66.49 ± 21.29	-0.876	0.383
Blood transfusion (U)	1.31 ± 1.16	1.29 ± 1.11	1.30 ± 1.13	0.116	0.908
Intraoperative liquid (ml)	1305.08 ± 302.53	1203.57 ± 266.26	1255.26 ± 289.98	1.906	0.059
Follow-up time (months)	10.98 ± 4.32	11.13 ± 3.92	11.11 ± 4.09	-0.184	0.854
Weight-bearing time (weeks)	2.71 ± 0.93	2.66 ± 1.01	2.69 ± 0.97	0.282	0.778
Clinical healing time (months)	5.83 ± 0.99	6.00 ± 0.92	5.92 ± 0.95	-0.955	0.342
Complications					
Deep vein thrombosis	22	23	45	0.173	0.678
Superficial infection	0	1	1	0.001	0.979
Revision	0	0	0		
Mortality	0	0	0		
HHS scores	83.02 ± 13.89	86.04 ± 3.39	84.47 ± 10.53	-1.554	0.123
Quality of reduction					
Excellent	32	35	67	0.875	0.646
Acceptable	25	19	44		
Poor	2	2	4		

## Data Availability

The [sav] data used to support the findings of this study are available from the corresponding author upon request.

## Abbreviations

PFNA: Proximal femoral nail antirotation
HHS: Harris Hip scores
VAS: Visual analogue scale.
